# Base calling for high-throughput short-read sequencing: dynamic programming solutions

**DOI:** 10.1186/1471-2105-14-129

**Published:** 2013-04-15

**Authors:** Shreepriya Das, Haris Vikalo

**Affiliations:** 1 Electrical and Computer Engineering Department, The University of Texas at Austin, Austin, Texas 78712, USA

## Abstract

**Background:**

Next-generation DNA sequencing platforms are capable of generating millions of reads in a matter of days at rapidly reducing costs. Despite its proliferation and technological improvements, the performance of next-generation sequencing remains adversely affected by the imperfections in the underlying biochemical and signal acquisition procedures. To this end, various techniques, including statistical methods, are used to improve read lengths and accuracy of these systems. Development of high performing base calling algorithms that are computationally efficient and scalable is an ongoing challenge.

**Results:**

We develop model-based statistical methods for fast and accurate base calling in Illumina’s next-generation sequencing platforms. In particular, we propose a computationally tractable parametric model which enables dynamic programming formulation of the base calling problem. Forward-backward and soft-output Viterbi algorithms are developed, and their performance and complexity are investigated and compared with the existing state-of-the-art base calling methods for this platform. A C code implementation of our algorithm named Softy can be downloaded from https://sourceforge.net/projects/dynamicprog.

**Conclusion:**

We demonstrate high accuracy and speed of the proposed methods on reads obtained using Illumina’s Genome Analyzer II and HiSeq2000. In addition to performing reliable and fast base calling, the developed algorithms enable incorporation of prior knowledge which can be utilized for parameter estimation and is potentially beneficial in various downstream applications.

## Background

Technological advancements in sequencing technologies have enabled fast sequencing of millions of reads at a rapidly reducing cost. Sequencing-by-synthesis, including Illumina’s platforms based on reversible terminator chemistry and 454’s pyrosequencing platforms, is taking us closer to affordable routine sequencing tasks. However, inherent uncertainties in the ensemble-based sequencing-by-synthesis, along with data acquisition noise, present a major bottleneck in the quest for reads that are as long and reliable as those provided by the conventional Sanger sequencing method.

Sequencing-by-synthesis on Illumina’s platforms typically involves the following steps. First, multiple copies of the genome being sequenced are broken into short fragments in a random fashion, followed by ligation of sequencing adapters to the fragments. In the next phase, the DNA sample is introduced into one of the 8 lanes (or 16 flow cells for HiSeq) split into multiple tiles each containing a lawn of primers covalently bonded to the surface to generate clonal clusters of the captured forward and reverse DNA strands. In particular, captured strands hybridize to neighboring primers to form so-called U-shaped bridges, followed by the process of bridge amplification which is repeated ≈ 35 times to generate clusters containing ≈ 2000 molecules. In the final stage, “sequencing”, fluorescently labeled reversible terminators (different color for each base) are introduced and incorporated into the complementary strands of the DNA templates. Imaging of the fluorescently labeled clusters is followed by cleavage and unblocking of the incorporated nucleotides, and the labeled reversible terminators are added anew to proceed with synthesis of the complementary strands.

Images acquired at the end of each sequencing cycle are processed, and the resulting signals are analyzed to determine the type of nucleotides incorporated into the complementary strands. The problem of inferring the order of nucleotides in a template from the noisy signals is referred to as *base calling*. For base calling, Illumina uses its in-house software *Bustard*. Fundamentally, Bustard attempts to reverse the effects of various uncertainties on the signal and then makes base calls. While it is computationally very fast, the error rates resulting from Bustard’s calls may be significantly improved by more sophisticated algorithms [1].

Several base calling strategies for the Illumina platform have been proposed in recent years such as [2-4]. In [5], a parametric model of the sequencing-by-synthesis process was proposed, and a Monte Carlo method for base calling was presented. While having better performance than Bustard, this scheme is computationally intensive and impractical for processing tens of millions of reads generated by today’s sequencers. Kao and Song 2010 [6], the follow-up paper, suggested a modification leading to a computationally feasible base calling albeit with slight degradation in performance compared to [5]. In [7], an algorithm achieving significant improvement in speed at the cost of a minor deterioration of base calling error rate as compared to [5] was presented.

Following a simplification of the parametric model of Illumina’s sequencing-by-synthesis platform proposed in [5], in this paper we present a formulation of the base calling problem amenable to being solved by dynamic programming methods. In particular, we derive the forward-backward and soft-output Viterbi algorithm (SOVA) for solving the base calling problem. The performance of the proposed algorithms is demonstrated on experimental reads acquired from Illumina’s Genome Analyzer II and HiSeq2000 and compared with several recent base calling techniques.

## Methods

In this section, we present the mathematical model that leads to the dynamic programming formulation of the base calling problem, and present algorithms for base calling and parameter estimation.

### Comprehensive model of sequencing-by-synthesis in Illumina’s platforms

A detailed mathematical model of Illumina’s platforms which describes various sources of uncertainty in the sequencing process and data acquisition step was introduced in [5]. For the sake of self-contained presentation, we briefly review this model here while omitting details for brevity.

The 4-dimensional observation vector *Y*_*i*_ (intensity) acquired in the *i*^*th*^ cycle (*i*=1,2,…,*N*) of the sequencing-by-synthesis process can be expressed as

(1) $$\begin{array}{rrr} Y_{i} & ∼N(KX_{i},\Sigma_{i})\;\;\;\;\;i=1,\; & \;\\ Y_{i}|Y_{i-1} & ∼N(KX_{i}+\alpha Y_{i-1},\Sigma_{i})\;i=2,\mathrm{....}\;N,\; \end{array}$$

where **K** is the 4×4 cross-talk matrix quantifying overlap of the emission spectra of the four fluorescent tags used to label nucleotide bases, *α* is the parameter accounting for empirically observed signal leakage between consecutive cycles, *X*_*i*_ is the signal generated in the *i*^*th*^ cycle, and $\Sigma_{i}=∥X_{i}{∥}_{2}^{2}\Sigma$ is the variance describing multiplicative measurement noise that primarily originates from fluctuations in **K**. The generated signal, *X*_*i*_, is affected by *phasing* and *pre-phasing*. In an ideal setting, addition of the four terminating base nucleotides during the sequencing step should lead to a single base getting incorporated into each of the complementary strands. However, nucleotide incorporation is not perfect and phasing (when no base is incorporated) or pre-phasing (when more than 1 base is incorporated) may occur. These effects are modeled probabilistically: it is assumed that no base is incorporated with probability *p*_*ii*_, while with probability *p*_*cf*_ more than 1 base is incorporated. For the sake of tractability of the final model and practical feasibility of base calling, we assume that at most two bases may be incorporated into a complementary strand in a single cycle. Define an (*L*+1)×(*L*+1) transition matrix **P** with entries

(2) $$P_{i,j}=\left\{\begin{array}{cc} P_{\text{ii}} & \;\text{if}\;\;j=i,\\ \left(1-P_{\text{ii}})(1-P_{\text{cf}}\right) & \;\text{if}\;\;j=i+1,\\ P_{\text{cf}}(1-P_{\text{ii}}) & \;\text{if}\;\;j=i+2,\\ 0 & \;\text{otherwise}, \end{array}\right)$$

where *P*_*i*,*j*_ is the probability of a complementary strand extending from length *i* to length *j* in any given cycle. Then the signal generated in the *i*^*th*^ cycle, *X*_*i*_, can be expressed as

$$X_{i}=\lambda_{i}Z_{i}=\lambda_{i}{\left(SE^{T}\right)}_{i},$$

where **S** is a 4×*L* matrix (The *i*^*th*^ column of **S**, *S*_*i*_, has all zero entries except for one indicating the base in the *i*^*th*^ position of the template. We follow the convention where the first component corresponds to *A*, the second to *C*, the third to *G* and the fourth to *T*) representing the template sequence of length *L*, **E** is an *N*×*L* matrix having entries *E*_*i*,*j*_=[**P**^*i*^]_0,*j*_ equal to the probability that the length of a strand after *i* cycles is *j*, and *λ*_*i*_ is a random variable describing empirically observed signal decay caused by the DNA loss due to primer-template melting, digestion by enzymatic impurities, DNA dissociation and misincorporation,

(3) $$\lambda_{i}|\lambda_{i-1}∼N((1-d)\lambda_{i-1},{\left(1-d\right)}^{2}\lambda_{i-1}^{2}\sigma^{2}),$$

where *d* is a constant droop factor over all cycles and all reads and *σ* is the standard deviation of *λ*_*i*_.

#### Illumina’s base calling approach

Prior to base calling, Bustard (Illumina’s base calling software) estimates cross-talk using signals generated by synthesizing the first 2 bases of all reads, evaluating entries of **K** as the median of the estimates obtained using individual read signals. Bustard then infers **X** by inverting **K** and multiplying it with **Y**. Next, it calculates a tile-wide average scalar ${\bar{X}}_{i}=\sum X_{j,i}$ and renormalizes the signal by multiplying *X*_*i*_ by ${\bar{X}}_{1}/{\bar{X}}_{i}$. This procedure corrects for the signal droop. Matrix **E** is estimated from the first 12 bases, inverted and multiplied by the normalized *X*_*i*_ values. This compensates for phasing/prephasing. Finally, for each cycle, base calling is performed by selecting the base inferred as having the highest corrected signal.

#### Parameter estimation and basecalling approach of BayesCall and naiveBayesCall

BayesCall relies on the comprehensive model reviewed in this section to perform base calling and significantly reduce error rates compared to Bustard. However, it suffers from two major computational bottlenecks. First, the lack of a closed form expression for the solution to the E-step of the EM algorithm used for parameter estimation necessitates a computationally intensive numerical optimization. Hence, the parameter estimation stage is time consuming, requiring ≈25 minutes per iteration on an 8 core machine. Consequently, BayesCall performs a single parameter estimation step that uses reads from all the tiles in a lane to generate a single set of parameters for the entire lane. Detailed analysis of BayesCall and naiveBayesCall error rates indicates that using a single set of parameters for an entire lane results in serious performance degradation for tiles where the parameters significantly differ from the ones used by the base calling algorithms (data not shown). Moreover, base calling in BayesCall is performed by relying on simulated annealing. Being a computationally intensive algorithm, the times for base calling via simulated annealing are prohibitively high. In order to overcome this issue, in the follow-up paper [6], the authors propose a simplified heuristic which reduces base calling times to ≈6 hours per lane with a small reduction in performance compared to BayesCall. However, since the parameter estimation step used by naiveBayesCall is the same as the one used by BayesCall, tiles with parameters which significantly differ from the single parameter set computed for the entire lane have very small performance improvements over Bustard.

### Our model refinements for fast tractable base calling

While providing detailed description of various sources of uncertainty, mathematical model of the sequencing process overviewed in the previous section leads to computationally demanding base calling algorithms. To simplify the model and enable practically feasible base calling, we approximate *λ*_*i*_ by its mean. Such an approximation is justified by the analysis of experimental data which shows that the coefficient of variation (ratio of the standard deviation to the mean) of *λ*_*i*_ in (3) is small (typically below 0.1 for early cycles and below 0.06 in the latter ones) [7]. On the other hand, it is desirable that the model allows variations in the droop factor from one cycle to another. Therefore, we describe the decay as $\lambda_{i}=\lambda \overset{i}{\underset{j=2}{\prod }}(1-{\bar{d}}_{j})$, where *λ* denotes a read-dependent transduction coefficient mapping synthesis events to the generated signal intensity, and ${\bar{d}}_{j}$ denotes cycle-dependent droop factors.

Note that the signal generated in the *i*^*th*^ cycle of the sequencing step, (**SE**^*T*^)_*i*_, can be expressed as

(4) $${\left(SE^{T}\right)}_{i}=\overset{L}{\underset{j=1,j\neq i}{\sum }}\beta_{i,j}S_{j}+(1-\overset{L}{\underset{j=1,j\neq i}{\sum }}\beta_{i,j})S_{i},$$

where *β*_*i*,*j*_s are dependent on *p*_*ii*_ and *p*_*cf*_. Based on the initial parameter estimates obtained using Monte Carlo methods, we observe that *p*_*ii*_ is very small. It is also observed that if we choose to retain only those terms in a given row of **E** that are at least 10*%* of the maximum entry, there is at most one base ahead of the tested base that contributes significantly to the signal *X*_*i*_. Consequently, we may approximate (**SE**^*T*^)_*i*_ in (4) as

(5) $${\left(SE^{T}\right)}_{i}\approx (1-\beta_{i,i+1})S_{i}+\beta_{i,i+1}S_{i+1},$$

where *β*_*i*,*i*+1_ is a cycle-dependent parameter which allows us to essentially approximate the nonlinear dependence of **E** on *p*_*cf*_ and hence facilitate efficient base calling.

**Figure 1:**
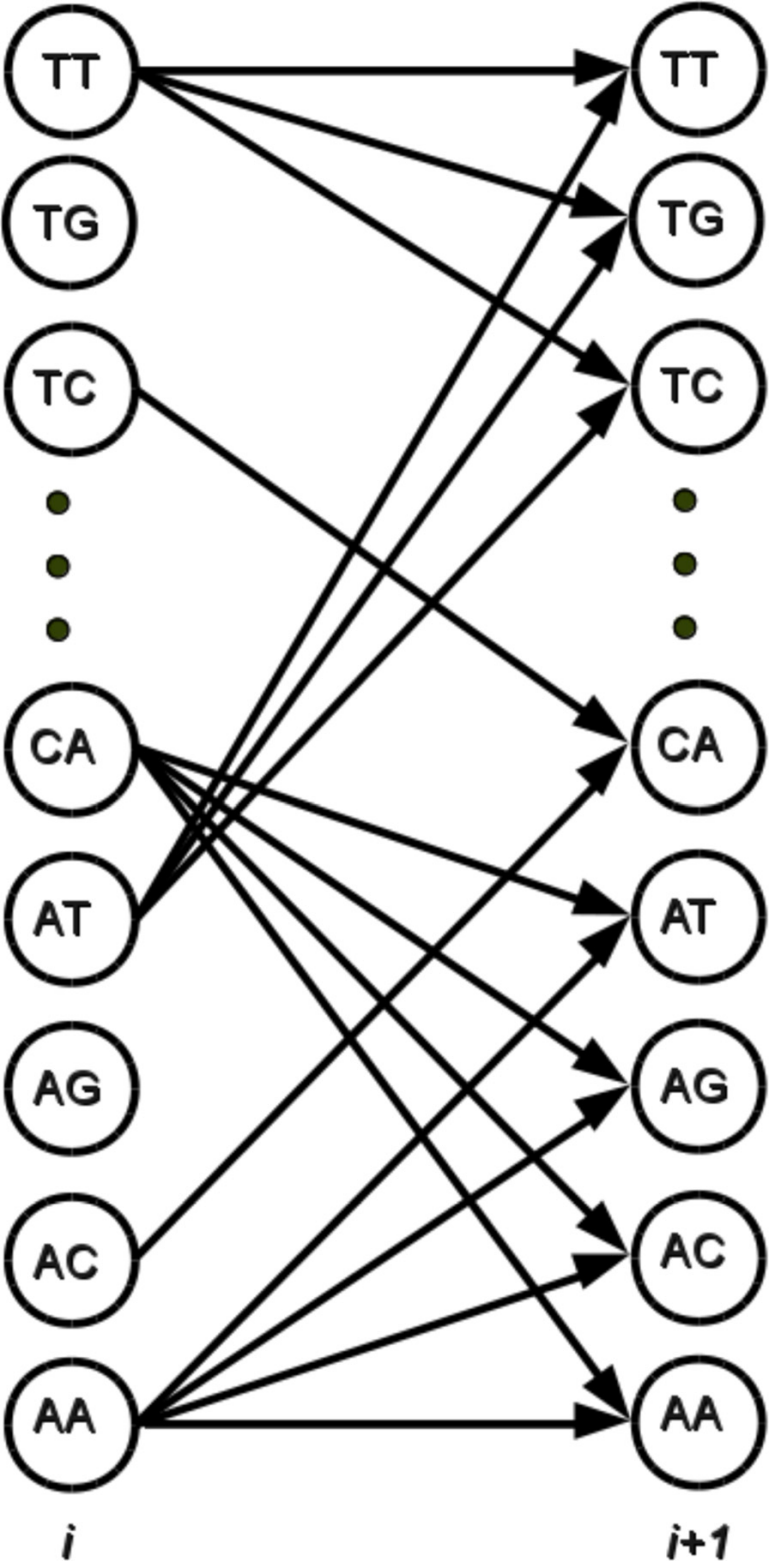
**The 16 state trellis illustration of the transitions between states in the*i***^***th ***^**and (*i+1*)**^***th ***^**stage of the trellis.** The figure shows 8 out of the possible 16 states along with all valid transitions between them.

For any given cycle *i*, the intensity of the signal in (5) is a function of *S*_*i*_ and *S*_*i*+1_. Such a finite memory approximation enables search for the optimal path *S*_1_,*S*_2_,…,*S*_*L*_ using dynamic programming principles. Graphically, this can be interpreted as the search on a 16-state trellis (The number of states needs to be increased if the parameters *p*_*ii*_ and/or *p*_*cf*_ are large, or if longer reads need to be called), where the states at the *i*^*th*^ stage of the trellis represent all possible pairs of bases in the *i*^*th*^ and (*i*+1)^*th*^ position of a read. We denote the states of the trellis by *T*_*i*_, where *i* is the cycle number. The states can take one of 16 possible values in the set {*AA*,*AC*,*AG*,…,*TT*}, 1≤*j*≤16. Note that not all state transitions are feasible. In particular, a transition from a state in cycle *i* to a state in cycle *i*+1 is valid if the second symbol of the state in cycle *i* is the same as the first symbol of the state in cycle *i*+1. Figure 1 illustrates two consecutive stages of the trellis. For the sake of tractability of the illustration, only 8 of the possible 16 states are shown. Arrows indicate valid transitions between the states that are included in the illustration.

#### Final model

Based on the discussion in the preceding section, the final model (for the *l*^*th*^ window) is of the form

(6) $$\begin{array}{l} Y_{i}∼N(\lambda K_{i}X_{i},\lambda^{2}∥X_{i}{∥}_{2}^{2}\Sigma_{i})\;i=1\\ Y_{i}|Y_{i-1}∼N(\lambda \overset{i}{\underset{j=2}{\prod }}(1-{\bar{d}}_{j})K_{i}X_{i}+\alpha_{i}Y_{i-1},\\ \;(\lambda \overset{i}{\underset{j=2}{\prod }}{\left(1-{\bar{d}}_{j})\right)}^{2}∥X_{i}{∥}_{2}^{2}\Sigma_{i})\;\;\;i=2,\mathrm{....}\;N \end{array}$$

where *X*_*i*_=((1−*β*_*i*_)*S*_*i*_+*β*_*i*_*S*_*i*+1_), and *β*_*i*,*i*+1_ is relabeled as *β*_*i*_ for the simplicity of notation. Let us collect all the parameters into a vector ***Θ***. Given ***Θ***, *λ* and *Y*_1_,*Y*_2_,…,*Y*_*N*_, the goal of base calling is to determine *S*_1_,*S*_2_,…,*S*_*L*_. In our approach, we first obtain estimates of the parameters ***Θ*** using an unsupervised learning scheme. Then, posterior probabilities of *S*_*i*_ are determined using either forward-backward or soft-output Viterbi algorithm. Details of the proposed scheme follow.

### Parameter estimation

We infer parameters of the mathematical model (6) by relying on an unsupervised estimation scheme. Unsupervised estimation need not be aided by a reference genome nor does it require analyzing a known sequence in a control lane. Our scheme also has the advantage of being implemented as an online, as opposed to a batch, algorithm. This allows parameter estimation (and base calling) of a previous window to be performed while the experiment is still in progress, resulting in smaller latency between the end of the run and basecalling results. In particular, we employ the online expectation-maximization (EM) algorithm [8] which relies on a training set of *R*=250 reads randomly selected across a tile. The optimization problem that the EM algorithm solves in an iterative fashion can be stated as

(7) $$\Theta_{n}=\arg\underset{\Theta }{\max}E_{(\lambda ,S)|\Theta_{n-1}}[\log P(S,\lambda |Y,\Theta )],$$

where the scalar coefficient *λ* and the template sequence matrix **S** are latent variables, $\Theta =\{\alpha ,\beta ,K,\Sigma ,{\bar{d}}_{j}\}$ is the set of parameters which need to be determined, and logP(S,λ|Y,Θ) denotes the log-posterior function. In the absence of any prior information, this is also the log-likelihood function. The expectation in (7) needs to be evaluated with respect to *λ* and **S** given the current estimates of ***Θ***.

The results from [5] indicate that base calling may be significantly improved by allowing the parameters to be cycle dependent. We observe the same and thus divide a sequencing run into windows of length *W*=6, and estimate model parameters window-by-window. Parameters for window *l* are initialized using the values of the parameters estimated in the previous window, *l*−1. To prevent over-fitting, (7) is optimized over two windows, *l* and *l*+1, and the resulting ***Θ*** is used as the set of parameters for window *l*. A window length *W*=6 was found to be short enough to capture time variations in the parameters and still maintain run times of the parameter estimation and base calling low.

#### Initialization for the first window

The EM algorithm requires initialization of the parameters in the first time window. We can reliably call the first two bases in a template by simply identifying the channels having the largest signal in the first two test cycles from which an initial estimate of **K** is obtained. As done by Bustard, multiple estimates of the columns of **K** can be computed from the first two signals of each read in a tile, and then the median of all these estimates can be used to provide an initial estimate $\hat{K}$. Subsequently, we find the mean of each column and add this to the diagonal entries. We then use the inverse of the resulting matrix to call bases again and iteratively refine the estimates of the entries of the matrix. The number of iterations is set to 5.

Given $\hat{K}$, an empirical estimate of *σ* is obtained by computing the difference between the intensity vector and the column of the cross talk matrix that corresponds to the called base. The covariance matrix ${\hat{\Sigma }}^{l}$ is computed from this for each read. Finally, the estimate $\hat{\Sigma }$ is formed as the median of ${\hat{\Sigma }}^{l}$. Parameters *α*_*i*_ and *β*_*i*_ are negligible in the early cycles and are initialized as zeros. To estimate droop coefficients ${\bar{d}}_{i}$, we start by calculating ${\hat{K}}^{-1}Y_{i}^{l}$ for each read and summing up the resulting vector elements to obtain the total signal $x_{i}^{l}$ acquired in the *i*^*th*^ cycle. The droop for the *i*^*th*^ cycle is then calculated as ${\hat{\bar{d}}}_{i}^{l}=x_{i}^{l}/x_{i-1}^{l}$ for each individual read. Finally, the median value of all ${\hat{\bar{d}}}_{i}^{l}$ is chosen as the initial value of ${\bar{d}}_{i}$. Details of this step are omitted and the interested reader is referred to [7].

#### E-step for the first window

The E-step requires finding the expectation of the log-likelihood function in (7) over *λ* and **S**. Closed form expressions are not available, while the numerical Monte-Carlo methods are computationally prohibitive in practice. As an alternative, we rely on Bustard’s approach to call sequences in the training set and use the resulting ${\hat{S}}^{k}$, 1≤*k*≤*R*, to approximate the expectation with respect to **S**. In particular, we approximate the objective of maximization (7) by

(8) $$\begin{array}{ll} O=-\sum_{k=1}^{R}E_{\lambda^{k}}\left[\sum_{i=(l-1)W+1}^{(l+1)W}\frac{1}{2}L\left(\lambda^{k},,,{Ŝ}_{i}^{k},,,\Theta_{l}\right)\right], & \; \end{array}$$

where

(9) $$\begin{array}{l} L(\lambda^{k},{Ŝ}_{i}^{k},\Theta_{l})=\text{logdet}{\left(\lambda^{k}\right)(\overset{i}{\underset{j=2}{\prod }}\left(1,-,{\bar{d}}_{j},)\right)}^{2}∥X_{i}{∥}^{2}\Sigma_{i})\\ +\frac{{\left(\bar{Y_{i}^{k}}\;-\;\lambda^{k}\overset{i}{\underset{j=2}{\prod }}(1\;-\;{\bar{d}}_{j})K_{i}X_{i}^{k}\right)}^{T}\Sigma_{i}^{-1}(\bar{Y_{i}^{k}}-\lambda^{k}\overset{i}{\underset{j=2}{\prod }}(1-{\bar{d}}_{j})K_{i}X_{i}^{k})}{{(\lambda^{k}\overset{i}{\underset{j=2}{\prod }}(1-{\bar{d}}_{j}))}^{2}{∥X_{i}∥}^{2}}, \end{array}$$

and ${\bar{Y}}_{i}=Y_{i}$ for *i*=1 and ${\bar{Y}}_{i}=Y_{i}-\alpha Y_{i-1}$ for *i*>1,*i*<=*N*. The superscript *k* is an index of a read in the training set and ranges from 1 to *R*. Then the expectation over *λ*^*k*^ in (8) is evaluated numerically via importance sampling, leading to an approximation of the objective function

(10) $$\begin{array}{ll} O\approx -\frac{1}{2}\overset{R}{\underset{k=1}{\sum }}\overset{(l+1)W}{\underset{i=(l-1)W+1}{\sum }}\overset{N_{\text{IS}}}{\underset{j=1}{\sum }}w_{j,k}L(\lambda_{j}^{k},{Ŝ}_{i}^{k},\Theta_{l}), & \; \end{array}$$

where *w*_*j*,*k*_ denote normalized weights of *N*_*IS*_=500 samples $\lambda_{j}^{k}$ generated for each read in the training set from the Gaussian distribution $N({\hat{\lambda }}^{k},0.1)$ (such a choice of sampling distribution for $\lambda_{j}^{k}$ is suggested by the analysis of experimental data). The mean of the sampling distribution for each read in the training set, ${\hat{\lambda }}^{k}$, is obtained by maximizing the log-likelihood function (9) given the current estimates of the parameters ***Θ*** and base calls ${\hat{S}}^{k}$.

#### M-step for first window

The objective function in (9) is separately differentiable and convex over each of the parameters in ***Θ*** except *β*. To optimize it, we rely on a cyclic co-ordinate descent scheme which rotates among the components of ***Θ***. To find *β*, we employ a grid search. The co-ordinate descent is terminated when the ratio of the change in the value of the objective function to the value of the objective function in a previous iteration is less than *ε* =0.003. We use a similar stopping criterion for termination of the expectation-maximization algorithm.

#### E-step for subsequent windows

Due to phasing effects and other imperfections affecting generated and measured signal, using Bustard’s calls to approximate expectation of the log-likelihood function as in (8) fails to provide reliable parameter estimates in subsequent windows. On the other hand, numerical evaluation of the objective function in (7), $E_{(\lambda ,S)|\Theta_{n-1}}[\log P(S,\lambda |Y,\Theta )]$, as we already argued in this section, is computationally prohibitive in practice. To facilitate practically feasible evaluation of the E-step for windows *l*>1, for each read in the training set we approximate the transduction coefficient *λ*^*k*^ by its mean, ${\hat{\lambda }}^{k}$, and replace the objective function by

(11) $$\overset{R}{\underset{k=1}{\sum }}\overset{M}{\underset{i=1}{\sum }}P(S_{i}|Y^{k},{\hat{\lambda }}^{k},\Theta )\log(P(S_{i}|Y^{k},{\hat{\lambda }}^{k},\Theta )),$$

where ${\hat{\lambda }}^{k}$ is obtained by maximizing the log-likelihood function (9) given the parameters inferred in the (*l*−1)^*st*^ window. Posteriori probabilities $P(S_{i}|Y^{k},{\hat{\lambda }}^{k},\Theta )$ needed to evaluate expression (11) can be found from the state posteriori probabilities. For instance, posteriori probability that the *i*^*th*^ base is A is

(12) $$P(S_{i}^{T}=[1\;0\;0\;0]|Y^{k},{\hat{\lambda }}^{k},\Theta )=\overset{4}{\underset{j=1}{\sum }}P(T_{i}=t_{j}|Y,{\hat{\lambda }}^{k},\Theta ),$$

where $t_{j}\in \{\text{AA},\text{AC},\text{AG},\text{AT},\text{CA},\ldots ,\text{TT}\}$, 1≤*j*≤16. Clearly, we need to find $P(T_{i}=t_{j}|Y,{\hat{\lambda }}^{k},\Theta )$. For this, we turn to dynamic programming ideas – in particular, the forward-backward and soft-output Viterbi algorithms.

#### Forward-backward algorithm

Denote the transition probability from state *k* in the *i*^*th*^ stage to state *l* in the (*i*+1)^*th*^ stage of the trellis by $a_{\text{kl}}=P(T_{i}=t_{k}|T_{i+1}=t_{l})$. If no prior information about transition probabilities is available, we will assume that the valid transitions are equally likely. Moreover, note that the state priors may be computed from the symbol priors, if those are available. For instance, prior for the state *T*_*i*_=*AC* can be found as the product of the priors for $S_{i}^{T}=[1\;0\;0\;0]$ and $S_{i+1}^{T}=[0\;1\;0\;0]$. Let $f_{l}(i)=P(Y_{1},Y_{2},\ldots ,Y_{i},T_{i}=t_{l})$ denote the so-called forward probabilities, and $b_{l}(i)=P(Y_{i+1},\ldots ,Y_{M}|T_{i}=t_{l})$ denote the backward probabilities. Moreover, let $e_{l}(Y_{i})=P(Y_{i}|T_{i}=t_{l},\lambda ,\Theta )$ denote emission probabilities. Then the recursion that computes forward probabilities can be stated as

$$f_{l}\left(i,+,1\right)=e_{l}\left(Y_{i+1}\right)\overset{16}{\underset{k=1}{\sum }}f_{k}\left(i\right)a_{\text{kl}},$$

while the backward recursion is given by

$$b_{k}\left(i\right)=\overset{16}{\underset{l=1}{\sum }}e_{l}\left(Y_{i+1}\right)a_{\text{kl}}b_{l}\left(i,+,1\right).$$

The recursions are initialized by setting *f*_0_(0)=1 and *b*_*k*_(*M*)=*a*_*k*,*e*_, where *a*_*k*,*e*_ denotes the probabilities of the terminating state as computed by the forward algorithm. Finally, the posterior probability is obtained as

$$P\left(T_{i},=,t_{k},|,Y,,,\lambda ,,,\Theta \right)=\frac{f_{k}(i)b_{k}(i)}{\overset{16}{\underset{j=1}{\sum }}f_{j}(i)b_{j}(i)},$$

for all 1≤*k*≤16, 1≤*i*≤*M*. In order to ensure that the finite size of the trellis does not adversely effect reliability of the computed probabilities, we add an extra 5 cycles in the calculations (i.e., we use *Y*_1_,…,*Y*_*M*+5_).

#### Soft output Viterbi algorithm

The forward-backward algorithm computes exact posteriori probabilities of the bases in a sequence. On the other hand, one can rely on various heuristics to obtain reasonably good approximations of posteriori probabilities while suffering only minor degradation in accuracy. Such heuristics include the soft-output Viterbi algorithm (SOVA), a modification of the Viterbi algorithm implemented on the same trellis we described in previous sections.

Let *v*_*k*_(*i*) denote the probability of the most likely state sequence which ends at *T*_*i*_=*t*_*k*_, i.e.,

$$v_{k}\left(i\right)=\underset{T_{1},\ldots ,T_{i-1}}{\max}P\left(Y_{1},,,\ldots ,,,Y_{i},,,T_{1},,,\ldots ,,,T_{i-1},,,T_{i},=,k\right).$$

Retaining the notation introduced for the description of the forward-backward algorithm, we can recursively compute *v*_*k*_(*i*) as

$$v_{l}(i+1)=e_{l}(Y_{i+1})\underset{k}{\max}a_{\text{kl}}v_{k}(i),$$

where the recursion is initialized by setting *v*_0_(0)=1, *v*_*k*_(0)=0 for all *k*>0. This recursion is at the core of the Viterbi algorithm, which then proceeds by backtracking through the optimal trellis path to determine the most likely sequence of states. The Viterbi algorithm, however, provides only the most likely sequence of states and does not find posteriori probabilities of the symbols. To this end, a soft-output variant of the Viterbi algorithm was proposed in [9]. SOVA traces back optimal path through the trellis and for each symbol (i.e., base) explores alternative paths that could have changed the decision of the Viterbi algorithm for that symbol. Cost metrics of the alternative paths are then used to approximate posteriori probabilities for the base under consideration. To allow computationally efficient procedure, we limit the length of deviation of the alternative paths from the optimal one to 3 edges. Note that it is necessary to normalize the posterior probabilities obtained in the described fashion. As we will demonstrate in the subsequent sections, the forward-backward algorithm achieves better base calling error rates than SOVA, but it does so at the cost of having reduced speed.

#### M-step for subsequent windows

The M-step for subsequent windows is very similar to the M-step for the first window. The only difference is that the objective function being maximized is now

(13) $$\begin{array}{ll} \;-\frac{1}{2}\sum_{k=1}^{R}\sum_{i=(l-1)W+1}^{(l+1)W}\sum_{j=1}^{4}P(S_{i}\;=\;s_{j}|Y^{k}\;,{\hat{\lambda }}^{k}\;,\Theta_{l})L(\lambda_{j},S_{i}\;=\;s_{j}^{k}\;,\Theta_{l}). & \; \end{array}$$

The optimization follows the same procedure as described for the first window.

*Updating*${\hat{\lambda }}^{k}$ - After each step of the EM algorithm used for estimating parameters in a given window, we make calls for $S_{i}^{k}$ (using outputs of either forward-backward or SOVA). The calls and the most recent parameters are then employed to update ${\hat{\lambda }}^{k}$ by maximizing the log-likelihood function (9). The updated value of ${\hat{\lambda }}^{k}$ is used by the EM algorithm in the next window.

### Base calling

Given ***Θ*** inferred by the EM algorithm and *Y*_1_,*Y*_2_,…,*Y*_*N*_, the goal of base calling is to determine *S*_1_,*S*_2_,…,*S*_*L*_, i.e., to find

(14) $${Ŝ}_{i}=\arg\underset{s_{j}}{\max}P(S_{i}=s_{j}|Y,\hat{\lambda },\Theta ),$$

where *s*_*j*_ can take values of unit vectors comprising three zeros and one non-zero entry equal to 1, and 1≤*j*≤4. Base probabilities $P(S_{i}=s_{j}|Y,\hat{\lambda },\Theta )$ can be calculated from the state probabilities of the trellis that we defined in the parameter estimation section, e.g.,

(15) $$P(S_{i}^{T}=[1\;0\;0\;0]\;)=\overset{4}{\underset{j=1}{\sum }}P(T_{i}=t_{j}|Y,\lambda ,\Theta ),$$

and so on. Note that these probabilities are also the ‘quality score’ assigned to the given basecall (more on quality scores in the next section). Clearly, we need to find posteriori probabilities $P(T_{i}=t_{j}|Y,\hat{\lambda },\Theta )$. For this, we again turn to the soft-output Viterbi and forward-backward algorithms that we described in the previous section.

Note that the value of $\hat{\lambda }$ used for base calling in window *l* is approximated by the value of *λ* which maximizes the log-likelihood function formed using ***Θ*** and ${Ŝ}_{i}$ from the previous window, *l*−1 (except in window *l*=1 where we use ${Ŝ}_{i}$ provided by Bustard). It is straightforward to show that this maximization entails solving the quadratic equation in *λ*

(16) $$\begin{array}{l} \overset{\text{lW}}{\underset{i=(l-1)W+1}{\sum }}{4\lambda }^{2}+\frac{{\left(K_{i}{\hat{X}}_{i}\right)}^{T}{\Sigma_{i}}^{-1}(\bar{Y_{i}})}{(\overset{i}{\underset{j=2}{\prod }}(1-{\bar{d}}_{j})){∥{\hat{X}}_{i}∥}^{2}}\lambda \\ \;-\frac{\bar{Y_{i}}{\Sigma_{i}}^{-1}\bar{Y_{i}}}{{\left(\overset{i}{\underset{j=2}{\prod }}(1-{\bar{d}}_{j})\right)}^{2}{∥{\hat{X}}_{i}∥}^{2}}=0, \end{array}$$

and choosing the positive solution as the value of $\hat{\lambda }$.

### Quality scores

Performance of various base calling algorithms can be compared by evaluating error rates that they achieve when applied to determining the order of nucleotides in a known sequence. In practical applications, where the sequence being analyzed is not known, we need to assess the confidence of a base calling procedure. To this end, *quality scores* provide information as to how reliable the corresponding base calls are. The quality scores that we assign to base calls are the posterior probabilities of the bases computed by the forward-backward/SOVA schemes. In particular, we use the posteriori probabilities of the bases computed according to (15) as the quality scores. In order to assess the ‘goodness’ of quality scores, we consider their discrimination ability [10,11]. The discrimination ability for a given error rate is obtained by sorting all bases according to their quality scores in descending order and finding the number of bases called before the error rate exceeds the predefined threshold.

## Results

### GAII

**Figure 2:**
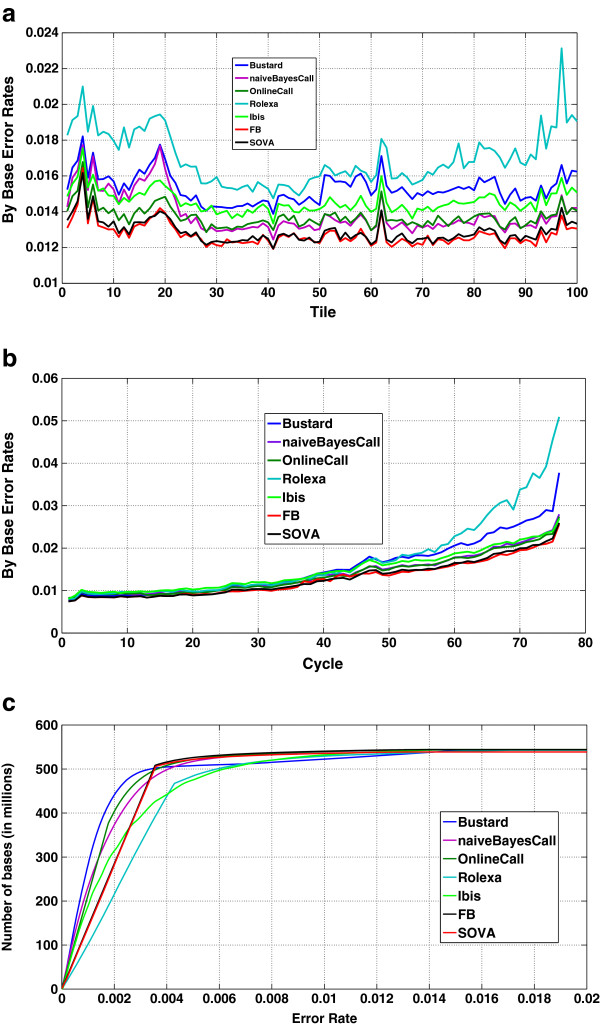
**(a,b,c) - Comparison of the different basecalling strategies under different performance metrics.** The figure shows **a**) Base calling error rates as a function of tile, **b**) Base calling error rates as a function of cycle number, **c**) Discrimination ability.

Performance of the forward-backward algorithm and SOVA is verified on a full lane data obtained by sequencing phiX174 ((EMBL/NCBI accession number J02482) bacteriophage using Illumina’s Genome Analyzer II which generates reads of length 76. After basecalling the lane by Bustard, naiveBayesCall, Rolexa, Ibis, forward-backward and SOVA, the calls were mapped onto the known reference sequence comprising 5386 bases. The optimal alignment is found using a Hamming distance metric. Reads that map with less than 30% errors are retained while reads having more errors are removed to ensure that there is no ambiguity in the alignment. This results in approximately 7 million reads and 550 million bases which are used to compare the performance of the considered basecalling schemes. Average error rates computed over the entire lane are compared in Table 1. Figure 2 shows the by tile error rates, by cycle error rates and the discrimination abilities of the different basecallers. Forward-backward algorithm and SOVA outperform all other schemes in terms of error rates and discrimination abilities.

**Table 1:** Comparison of error rates and speed for GAII

**Decoding strategy**	**Error rate**	**Running times**
FB	0.0128	400mins
SOVA	0.0129	300mins
OnlineCall	0.0137	30mins
naiveBayesCall	0.0139	1500mins
Ibis	0.0147	480mins
Bustard	0.0154	40mins
Rolexa	0.0171	720mins

A comparison of error rates and running times (per lane) for different base callers (note that Bustard’s running time is underestimated since it does not account for the parameter estimation step).

#### HiSeq

**Figure 3:**
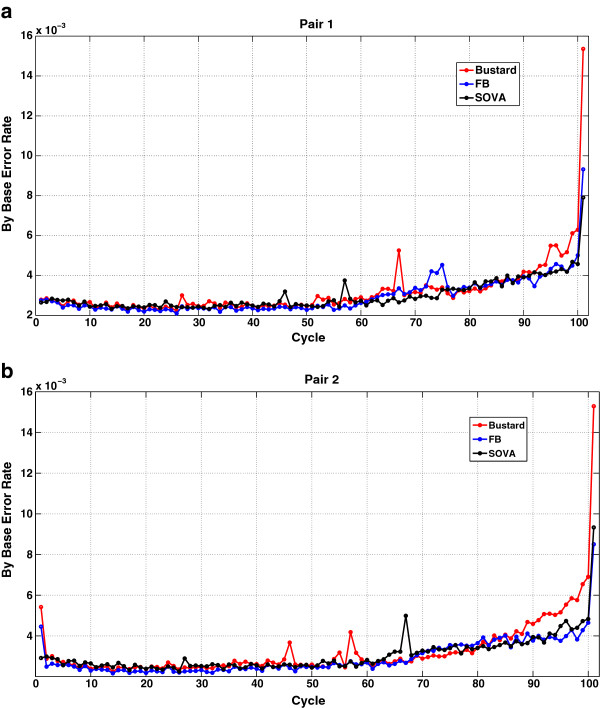
**(a,b) - Comparison of the different basecalling strategies for HiSeq2000.** The figure shows **a**) Base calling error rates as a function of cycle for the first pair, **b**) Base calling error rates as a function of cycle for the second pair.

Performance of the forward-backward algorithm and SOVA is verified on reads from E.coli (EMBL/NCBI accession number NC007779) using Illumina’s HiSeq2000 comprising of 100 cycle paired end data. The error rates for both pairs of reads are shown as a function of cycle number in Figure 3. Average error rates are compared in Table 2 for both SOVA and FB schemes. As can be seen, we improve on Bustard’s calls by 12.3 and 9.6*%* for the first and second pair respectively.

**Table 2:** Comparison of error rates for HiSeq

**Decoding strategy**	**Error rate (Pair 1)**	**Error rate (Pair 2)**
FB	0.0029	0.0029
SOVA	0.0029	0.0029
Bustard	0.0033	0.0032

A comparison of error rates for different base callers for HiSeq.

## Discussion

### Computational complexity

For each read, the most computationally expensive Bustard’s step is its correction of phasing effects. For both forward-backward algorithm and SOVA, we need to evaluate 16 objective functions for the states at each stage of the trellis. In order to avoid finite window effects, for each window of length 6 additional 5 cycles are included in the computations. Therefore, for a 76 cycle read, we need to evaluate 131×16 state values. Additional overhead due to combining these values requires mostly additions (when the algorithms are implemented in the log domain). naiveBayesCall on the other hand, performs matrix inversion of the same complexity as those performed by Bustard, followed by evaluation of 4×76×21 terms. The factor 21 arises due to the fact that naiveBayesCall needs to solve a quartic equation using a golden section search that requires 21 evaluations per base. Thus, forward backward and SOVA are ≈3 times faster than naiveBayesCall.

### Implementation and running times

We implemented our codes on an Intel i7 machine *@*3.07GHz using only a single core. With our codes written in C, it takes approximately 240 seconds to read in an intensity file, perform the parameter estimation step on 250 reads, call bases for the whole tile and write it in fastq format for the forward backward scheme and 180 seconds for SOVA. Processing an entire lane requires about 400 minutes and 300 minutes for FB and SOVA, respectively. naiveBayesCall, on the other hand, requires 19 hours just for its parameter estimation step while its basecalling takes 6 hours. Thus, our FB and SOVA implementations are 4 and 5 times faster than naiveBayesCall. Note that the run times of naiveBayesCall are reported for an implementation on a processor with 8 cores; it is expected that a parallel implementation of our algorithm would reduce the total running time by roughly 8 times. In addition, our proposed schemes would be able to almost instantaneously provide very high quality base calls to the end user since they are online (as opposed to batch) in nature. A comparison of the running times for processing an entire lane between the forward-backward algorithm and SOVA and the other basecallers is shown in Table 1.

### Improving error rates using supervised parameter estimation

Although the described parameter estimation procedure assumes no supervision, the proposed forward-backward and SOVA schemes allow incorporation of non-uniform priors that may improve accuracy of the inferred parameters and hence the overall base calling performance. Illumina platforms typically have a dedicated control lane comprising reads from a known reference. In such a case, it is possible to obtain priors by aligning the reads onto the reference and using them to improve the accuracy of the estimated parameters.

To this end, we utilize the calls from Bustard and align the reads onto the reference *phiX*174 genome using the same mapping criteria as described in the Results section. A basecall that is perfectly mapped to the reference is assigned a prior probability of 1, while in case of a mismatch the prior probabilities are split between the base suggested by the reference and the base called. If the reference is not very trustworthy, lower prior can be assigned to the base implicated by the reference. The described change requires very minor modification of the parameter estimation step. Table 3 shows the improvement obtained using the supervised scheme. Both forward-backward and SOVA schemes benefit marginally if the parameters are estimated in the supervised setting.

**Table 3:** Comparison of error rates for supervised and unsupervised schemes

**Decoding strategy**	**Error rate**
FB (unsupervised)	0.0125
SOVA (unsupervised)	0.0127
FB (supervised)	0.0124
SOVA (supervised)	0.0126

A comparison of error rates for supervised and unsupervised schemes on a single tile for GAII.

## Conclusion

We presented a formulation of the base calling problem on Illumina platforms that is amenable to being solved by dynamic programming methods, and proposed forward-backward and soft-output Viterbi algorithms for solving it. Base calling error rate performance of the proposed algorithms was demonstrated on experimental data to be superior to Illumina’s Bustard and several other publicly available base callers. The developed base callers are tested on data obtained by Genome Analyzer II and HiSeq2000 but the model, concepts, and algorithms should apply to other Illumina’s platforms as well. The developed schemes are online (as opposed to batch), scalable, and much faster than competing model-based base callers. In addition, they are capable of accounting for soft inputs (priors) and generating soft outputs (posteriors) – a feature we exploited to devise a supervised scheme for learning parameters of the sequencing model, and may further be useful in applications where prior knowledge about reads is available.

## Competing interests

The authors declare that they have no competing interests.

## Authors’ contributions

Algorithms and experiments were designed by S Das (SD) and Haris Vikalo (HV). Algorithm code was implemented and tested by SD. The manuscript was written by SD and HV. Both authors read and approved the final manuscript.
